# Flow-Based Three-Dimensional Co-Culture Model for Long-Term Hepatotoxicity Prediction

**DOI:** 10.3390/mi11010036

**Published:** 2019-12-27

**Authors:** Yoon Young Choi, Jin-I Seok, Dong-Sik Kim

**Affiliations:** Department of HBP Surgery & Liver Transplantation, Department of Surgery, Korea University College of Medicine, Seoul 02841, Korea; isteroo@naver.com (Y.Y.C.); ppm0113@naver.com (J.-I.S.)

**Keywords:** microfluidic, hepatic stellate cell, hepatotoxicity, spheroid, co-culture

## Abstract

We developed concave microwell arrays to establish a size-controllable 3-D co-culture liver model for in vitro drug toxicity testing, to predict hepatotoxicity. The interaction of hepatocytes with hepatic stellate cells (HSCs) was investigated by co-culturing primary 3-D hepatocyte spheroids and HSCs (heterosphere), using 3-D liver-on-a-chip. The effect of HSCs was investigated during spheroid formation; they were involved in controlling the organization of spheroidal aggregates and the formation of tight cell–cell contacts. Scanning electron microscopy (SEM) images showed that co-cultured spheroids with smoother surfaces in the flow chip aggregated more tightly and rapidly, compared to mono-cultured spheroids, until 13 days. Metabolic function analysis revealed that heterospheres secreted 40% more albumin and urea than hepatospheres on day 13. Additionally, an acetaminophen (AAP) and isoniazid (INH) concentration-dependent increase in CYP3A4 expression was detected in the 3-D cultures, and an increase in Lactate dehydrogenase (LDH) release after AAP and INH treatment was observed. CYP1A2, Mrp1 and UGT1A5 mRNA expression levels in the heterospheres and hepatospheres were evaluated from days 3 to 13. To examine the potential for toxicity testing in the flow-conditioned culture of the heterospheres, we evaluated cytotoxicity using the endpoint LDH release in the heterospheres and hepatospheres. IC_50_ values for AAP and INH after 24 h of exposure were calculated from the dose–response curves of the compounds. Flow-conditioned heterosphere culture results suggest that it may be suitable for long-term culture and cytotoxicity testing. Thus, our co-culture system closely resembles the in vivo environment and allows long-term in vitro hepatotoxicity prediction.

## 1. Introduction

The liver is the largest organ in the body and performs vital functions related to digestion, metabolism and the detoxification of xenobiotics [1]. The healthy liver is composed of a parenchymal compartment, hepatocytes and a non-parenchymal compartment comprising endothelial cells, Kupffer cells and hepatic stellate cells (HSCs) [2]. The hepatocytes constitute approximately 80% of the human liver, and are involved in protein synthesis and storage, carbohydrate transformation, cholesterol synthesis, and detoxification [3,4,5]. The HSCs constitute about 5–12% of the total cells in the normal liver and play a key role in hepatic fibrosis as the main secretory cells in the extracellular matrix proteins of the damaged liver [6,7]. In addition, HSCs are known to be involved in the regeneration of the liver and the production of mediators, such as hepatocyte growth factor (HGF), insulin-like growth factor (IGF) and transforming growth factor β1(TGF-β1) [8,9,10]. 

In the present day, various hepatocytes in vitro culture systems have been explored, including culture on membrane gels [11], co-culture systems [12], stacked culture [13] and three-dimensional (3-D) culture systems [14]. Among these, the primary 3-D culture of hepatocyte spheroids is essential to reconstruct functional hepatic tissues *in vitro*, and is advantageous in maintaining long-term hepatic functions for extended culture periods [15]. However, it is difficult to establish 3-D cultures capable of producing and maintaining hepatocyte spheroids, as necrosis may occur inside the core of large spheroids [16]. For in-depth studies of the liver under in vitro conditions, new cell culture models, which allow 3-D micro cell–cell formation and cell–cell interactions affecting the modulation of hepatocytes and HSCs, and facilitate the investigation of metabolic functions, are required. 

Previously, we developed a novel, microfluidic chip to investigate the flow effects on hepatocytes, mimicking the in vivo environment with portal flow [17]. In the present study, a cell suspension of hepatocytes and HSCs was applied to a spheroid-based 3-D artificial liver chip (ALC) with a perfusion function, at a ratio of 9:1 (hepatocytes:HSCs), in order to investigate the effects of HSCs on the viability and function of hepatocytes in flow culture. Compared to previous culture systems, this newly optimized system enables the construction of uniform and regular sized spheroids using 3-D concave microwells and allows continuous laminar flow for a longer time. The chip enables a continuous supply of oxygen and nutrients and removal of wastes, similar to that in the in vivo environment. We investigated the cell viability, liver-specific function, metabolic function and spheroid surface morphology. In addition to determining the effects of HSCs on these hepatocyte spheroids, we also performed toxicity tests on both spheroid’s models.

## 2. Materials and Methods 

### 2.1. Fabrication of Concave Chip with Flow

**Figure 1:**
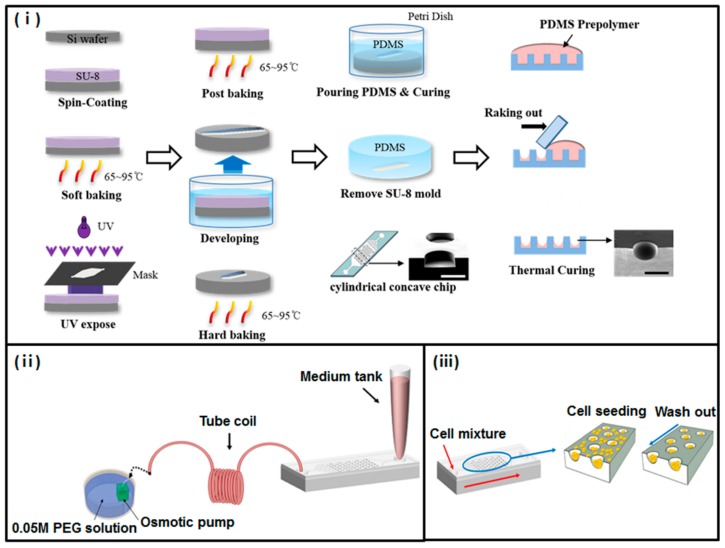
Fabrication of a concave microwell array polydimethylsiloxane (PDMS) plate. (**i**) Schematic diagram of concave microwell array made using the conventional soft-lithography procedure and meniscus of the PDMS pre-polymer. (**ii**) Experimental setup. Micropipette tips were used as inlet reservoirs. The osmotic pump was dipped into the polyethylene glycol (PEG) solution to generate the flow rate of the microsystem; the coiled tube was used as an outlet reservoir. (**iii**) Schematic process of cell seeding and docking within concave microwell structures. After gently aspirating cells that were not docked, the remaining cells docked within concave microwells.

The concave chip was fabricated using the surface tension of polydimethylsiloxane (PDMS) prepolymer, as we have previously described [18]; the detail of this process is presented in Figure 1. A basic structure PDMS chamber containing a 50-cylidrical-well array structure (diameter 500 μm; height: 400 μm) was prepared using a conventional soft lithography process. Onto the basic structure PDMS chamber, we poured a PDMS prepolymer, and allowed it to completely fill all cylindrical microwells. Then we racked out the prepolymer using slide glass. Each of the microwells formed a concave meniscus through surface tension when half of the PDMS prepolymer remained in these cylindrical microwells. The prepolymers remaining in microwells were polymerized on a 90 °C hot plate for 1 h. We prepared the top layer, which is a plane with inlet and outlet holes. Finally, the top and bottom layer bonded together, using oxygen plasma treatment. For the osmotic pump, PDMS cubic chambers (1 × 1 × 1 cm) with a cellulose membrane window (5 × 5 mm) were fabricated following a previously reported process [19]; the cellulose membrane was bonded to the PMDS chamber using PDMS prepolymer as the adhesive glue. Deionized water was used as a buffer solution, and polyethylene glycol (PEG; Sigma Aldrich; molecular weight = 2000) solution was used as a driving agent. The concentration of PEG solution was determined based on results from a previous study [17].

### 2.2. Isolation and Culture of Primary Hepatocyte and HSCs

Hepatocytes and hepatic stellate cells (HSCs) were isolated from 15 weeks adult male Sprague–Dawley rats (DBL, Seoul, South Korea) by a two-step collagenase perfusion procedure according to the method previously described [20]. Briefly, the perfused liver was dissected, suspended in the serum-free culture medium and filtered through 100 μm nylon mesh to remove the tissue debris. Then the cell suspension washed twice by centrifugation at 50× *g* for 5 min. At this time, cell pellets were used for hepatocyte isolation, the supernatant was used for HSCs isolation. Dead hepatocytes were removed by Percoll (Sigma Aldrich, MO, USA) gradient centrifugation (200× *g*, 15 min) at 4 °C, and viable hepatocytes were resuspended in 1,3-Bis(hydroxymethyl)-5,5-dimethylimidazolidine-2,4-dione (DMDM) culture medium supplemented with 20 mM HEPES (Sigma Aldrich, St. Louis, MO, USA), 25 mM NaHCO_3_, 30 mg/L-proline, 10% fetal bovine serum (FBS), 25 U/mL penicillin, 25 mg/mL streptomycin, 10 mg/mL gentamicin, 10 ng/mL epidermal growth factor (EGF), 50 ng/mL insulin, 10^−4^ M dexamethasone, 10 mM nicotinamide and 100 mM L-ascorbic acid. For the obtained HSCs, supernatants were centrifugation repeated in 50× *g* for 5 min until the pellet disappeared. Finally, centrifugation at 200× *g* for 10 min formed a pellet that contained the HSCs. HSCs were cultured in high-glucose DMEM containing 10% FBS, 50 U/mL penicillin, and 50 mg/mL streptomycin. HSCs were used for 5–7 after culture days. This study was approved by the Korea University Institutional Animal Care and Use Committee (KUIACUC-2013-130, 04 July 2013). All the procedures were performed in accordance with the guidelines of the IRB of Korea University. 

### 2.3. Formation of Hepatospheres and Heterospheres in the Concave Chamber

Before cell seeding, the concave microwells were coated with 3% (*w*/*v*) bovine serum albumin (BSA) for 3 h to prevent cell adhesion, after which concave microchips were rinsed three times with PBS. Then, a primary hepatocyte suspension (2 × 10^6^ cells/100 µL) was loaded into the microchannel using a micropipette, allowing the cells to be trapped within the concave microwells to generate hepatospheres. For heterosphere formation, a suspension of hepatocyte and HSCs (2 × 10^6^ cells/100 µL) at a 9:1 ratio was loaded using different sets of same type of microsystem. Most cells were evenly docked within the concave microwells. The cells were left in the incubator for 30 min without flow for stabilization within the microwells. After 30 min of cell seeding, using the gravity, less than 100 μL of culture medium was allowed to flow gently to remove the cells that did not dock within the microwells. The average count of cells in the system at this time point was 1 × 10^5^, and the estimate of cell count in each well was about 2 × 10^3^. Once the chips were enclosed with a flexible polyurethane tube and connected to the osmotic pump, the microfluidic device was transferred to a humidified CO_2_ incubator at 37 °C. Cell aggregation and spheroid formation were observed daily under a microscope. 

### 2.4. SEM

The spheroids were fixed with 2.5% glutaraldehyde in deionized water for 1 h; then, they were washed 3–5 times with deionized water. For secondary fixation, the spheroids were treated with in 1% osmium tetroxide with deionized water for 1 h. After dehydration in graded ethanol series (25–100%), the spheroids were immersed in tert-butyl alcohol for 30 min (three times) at room temperature and then freeze-dried until the tert-butyl alcohol was dehydrated at −70 °C. The spheroids were then mounted with graphite paste, coated with palladium alloy and viewed with a scanning electron microscope (JEOL Ltd., Tokyo, Japan). 

### 2.5. Immunofluorescence Staining

7 and 14 days cultured heterospheres and hepatospheres were taken out of the concave microwells and transferred to a 35 mm petri dish. Spheroids were washed with PBS once to remove the culture medium. Then spheroids were fixed with 4% paraformaldehyde (PFA) for 30 min at 4 °C and, permeabilized with phosphate-buffered saline containing 0.1% Triton X-100 for 20 min at room temperature. After washing with 0.1% BSA in PBS, the spheroids were blocked with Block Ace (Dainippon Pharma, Tokyo, Japan) for 30 min at 4 °C. Thereafter, the cells were incubated with mouse anti-albumin primary antibodies (Santa Cruz Biotechnology Inc., Santa Cruz, CA, USA) at 4 °C overnight. The cells were then rinsed with 0.1% BSA in PBS, followed by incubation at 4 °C for 90 min with Alexa Fluor 488-conjugated anti-mouse (Invitrogen) secondary antibodies, as appropriate. The cells were then incubated with 4’,6-diamidino-2-phenylindole (DAPI) for 5 min at room temperature before collecting confocal microscopic images (Olympus, Tokyo, Japan).

### 2.6. Cell Viability

Cell viability was assessed by incubating the spheroids with 50 mM Calcein-AM and 25 mg/mL ethidium homodimer-1 (Invitrogen, Carlsbad, CA, USA) in culture medium for 40 min at 37 °C, and then we examined the surfaces of the spheroids using an inverted fluorescence microscope (AxioVision 4, Zeiss, Oberkochen, Germany).

### 2.7. Functional Assessment

Albumin and urea secretion were analyzed by measuring the concentration of albumin and urea in the medium conditioned by the cultured spheroids. The spheroids were cultured in concave flow chips. After culturing for 1, 3, 5, 7, 9, 11 and 13 days, the medium collected in the coiled tube to the osmotic pump was analyzed for albumin and urea concentration. Then microsystems were replaced with fresh medium and a new coiled tube. 

The Luminescent P450-Glo CYP3A4 assay Kit (Promega, Madison, WI, USA) was used to measure the activities of the CYP3A4 in accordance with the manufacturer’s instructions. Briefly, both acetaminophen (AAP)-treated spheroids were incubated at 37 °C in culture medium supplemented with luminogenic substrate. After 4 h of incubation, the culture medium was transferred to the new 96-well opaque white plate and mixed with equal amounts of luciferin detection reagent to initiate the luminescent reaction. After 20 min of incubation at room temperature, luminescence was measured with a Multimode Plate (PerkinElmer, Waltham, MA, USA).

### 2.8. Reverse Transcriptase Polymerase Chain Reaction (RT-PCR)

The hepatospheres and heterospheres were cultured within the concave chips for 3, 5, 7, 9, 11 and 13 days *in vitro*, after which the cells were gently retrieved, and subsequently, resuspended in hepatocyte culture medium. Digestion in TRI-reagent was performed, followed by chloroform extraction and precipitation with isopropyl alcohol. cDNA was generated from purified RNA using reverse transcriptase (TAKARA, Shiga, Japan), according to the manufacturer’s instructions.

### 2.9. Preparation and Treatment of Drugs, Cytotoxicity Test

AAP (Sigma Aldrich, St. Louis, MO, USA) was made up as a 100 μM stock solution in PBS. Isoniazid (INH) (Sigma Aldrich, St. Louis, MO, USA) was made up as a 100 mM stock solution in dimethyl sulfoxide (DMSO). Then both reagents were diluted in culture medium to give an appropriate final concentration. Briefly, 3, 7 and 14 days-cultured spheroids were treated 0.5, 1, 2.5, 5, 10 µM AAP and 1, 2, 4, 6, 8 mM INH concentrations for 24 h. After incubation, the cell medium transferred into corresponding wells which were optically clear with a 96-well flat bottom plate. LDH activity was measured using a Cytotoxicity Detection Kit (MK401, TAKARA, Shiga, Japan) according to the manufacturer’s procedure. Finally, the OD at 490 nm with a reference wavelength of 690 nm for each sample was measured. LDH is a soluble cytosolic enzyme that is released into the culture medium following the loss of membrane integrity. LDH activity, therefore, can be used as an indicator of cell viability. The degree of cytotoxicity was expressed as the percentage of LDH release and was estimated in the following way:

(1) $$Cytoxicity\;\left(\%\right)=\;\frac{Esperimental\;Absorbance-Low\;Control}{High\;Control-Low\;Control}\;\times \;100$$

Inhibitor concentration 50% (IC_50_) has calculated by fitting the data to the log (inhibitor) vs. response equation. All data analyses were performed using GraphPad Prism version 8.0 (San Diego, CA, USA).

### 2.10. Statistical Analysis

Experiments for albumin and urea were conducted in five times independent experiments. Hepatocyte-specific gene expression assessment was conducted in triplicate. Metabolic activity and cytotoxicity tests were conducted with four times independent experiments. SPSS statistics 23.0 (IBM corporation, Armonk, NY, USA) was used for statistical analysis. Results are expressed as mean ± standard deviation (SD). A two-tailed unpaired student’s t-test was used to test significance between individual data sets as indicated. A *p*-value < 0.05 was considered statistically significant. 

## 3. Results

### 3.1. Morphological Observation and Viability of Spheroids Cultured in Concave Flow Chips

**Figure 2:**
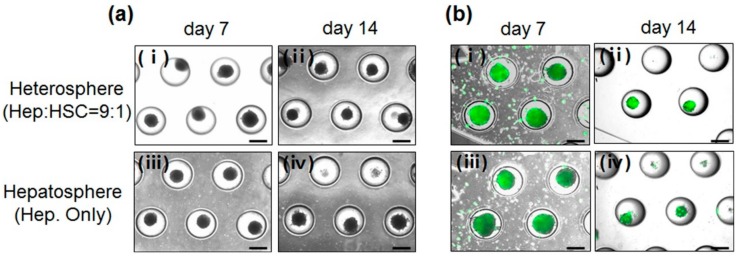
(**a**) Light microscopic images of spheroids formed in concave microwells. (**b**) Viability of cells in the hepatospheres cultured for (**i**) 7 and (**ii**) 14 days; heterospheres cultured for (**iii**) 7 and (**iv**) 14 days in the concave flow chip. Scale bar = 500 µm.

We observed that the mixture of hepatocytes and HSCs and the hepatocytes themselves, aggregated in the center of the concave microwell flow chip to form small spheroids (Figure 2a). The hepatic cells cultured in the concave flow chip were physically constrained and formed spheroids, which were homogeneous in size. 

**Figure 3:**
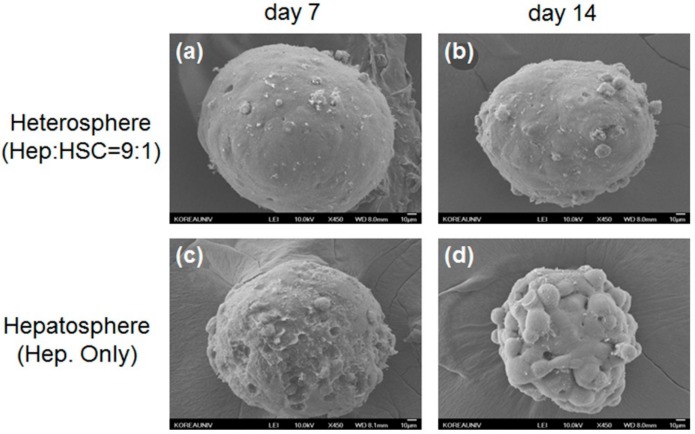
Scanning electron microscopy (SEM) images of heterospheres cultured for (**a**) 7 and (**b**) 14 days; hepatospheres cultured for (**c**) 7 and (**d**) 14 days. Scale bar = 10 μm.

These results showed the effect of the concave microwell arrays on spheroid formation and indicated that concave microwell arrays more effectively generate homogeneous heterospheres from co-cultured hepatocytes and HSCs. The cell viability of the heterospheres and hepatospheres formed in the concave flow chips after culturing for 7 and 14 days was approximately 95% (Figure 2b). As shown in Figure 3, the scanning electron microscopy (SEM) images clearly showed that the co-cultured spheroids with smoother surfaces in the flow chip aggregated more tightly and rapidly than the mono-cultured spheroids after 14 days of culture (Figure 3b). However, the mono-cultured spheroids did not maintain spheroidal shape after 14 days of culture (Figure 3d). The SEM images of the hepatospheres and heterospheres, cultured for 7 and 14 days in the concave flow chips, showed a greater degree of tight cell–cell contact in the heterospheres with smooth surfaces, compared to the hepatospheres. 

### 3.2. Functional Evaluation of Spheroids Culture in Concave Flow Chips

**Figure 4:**
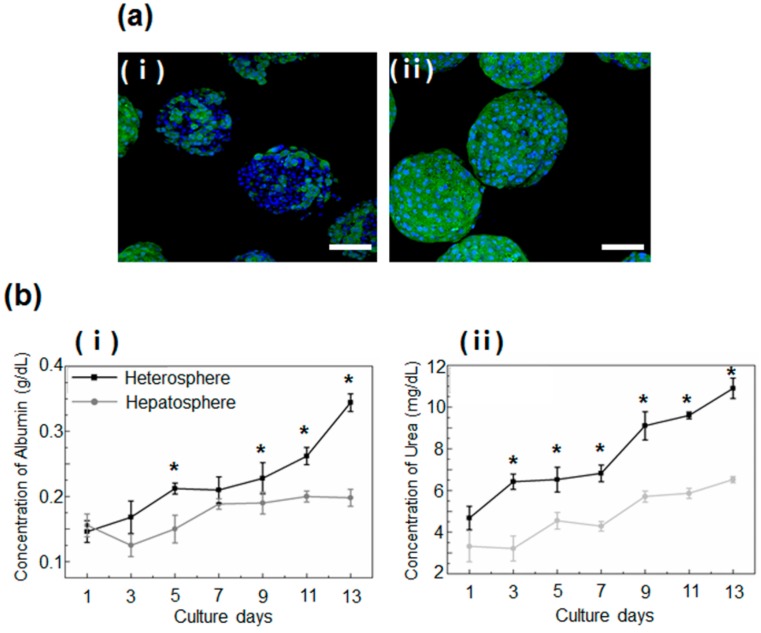
(**a**) Immunostaining for serum albumin (green) in the (**i**) hepatospheres and (**ii**) heterospheres cultured for 2 weeks. The nuclei were stained with DAPI (blue). Scale bar = 100 μm. (**b**) Analysis of the function of heterospheres and hepatospheres, measured in terms of the secretion of (**i**) albumin and (**ii**) urea. Data are represented as the mean ± standard deviation (SD) of five independent experiments. * *p* < 0.05 for hepatosphere vs. heterosphere.

The analysis of the function and metabolic activity of the hepatocyte spheroids in the heterospheres and hepatospheres performed by the immunostaining and measurement of the secreted albumin and urea concentration, showed significant differences between the two models. The heterospheres exhibited a greater degree of albumin production than the hepatospheres (Figure 4a), after 14 days of culture. 

This finding was reconfirmed by a quantitative analysis of albumin and urea secretion, which showed greater secretion of albumin in the heterospheres than in the hepatospheres. These data indicated an increase in the liver-specific functions of spheroids co-cultured with HSCs (Figure 4b).

### 3.3. Hepatocyte-Specific Gene Expression of Spheroids Culture in Concave Flow Chips

**Figure 5:**
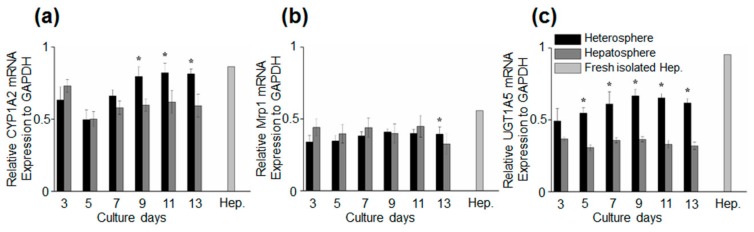
Time-course of marker expression for specific hepatocytes. The quantification of the relative gene expression of (**a**) CYP1A2, (**b**) Mrp1, and (**c**) UGT1A5. Data are represented as the mean ± standard error (SE) of three independent experiments. * *p* < 0.05.

We analyzed the hepatocyte-specific function of spheroids under flow culture by evaluating the expression of gene markers from day 3 to 13. We analyzed the mRNA expression levels of CYP1A2 (a marker for the metabolism of xenobiotics in the body), Mrp1 (a marker of multi-drug resistance), and UGT1A5 in the spheroids on days 3 to 13 and fresh hepatocytes (Figure 5). An increase in the mRNA expression levels of all the markers was observed in the heterospheres on day 13. The mRNA level of UGT1A5 was higher in the heterospheres than in the hepatospheres during the experiment, which indicates that the HSCs in the heterospheres affected hepatocyte function in the flow culture. Furthermore, the CYP1A2 expression in heterospheres was significantly higher than that in the hepatospheres under flow culture conditions. These results demonstrated that spheroids cultured with HSCs maintain their functionality better than spheroids consisting of a single hepatocyte.

### 3.4. Drug Induced Hepatotoxicity and Metabolic Function Evaluation of Spheroids Culture in Concave Flow Chips

**Figure 6:**
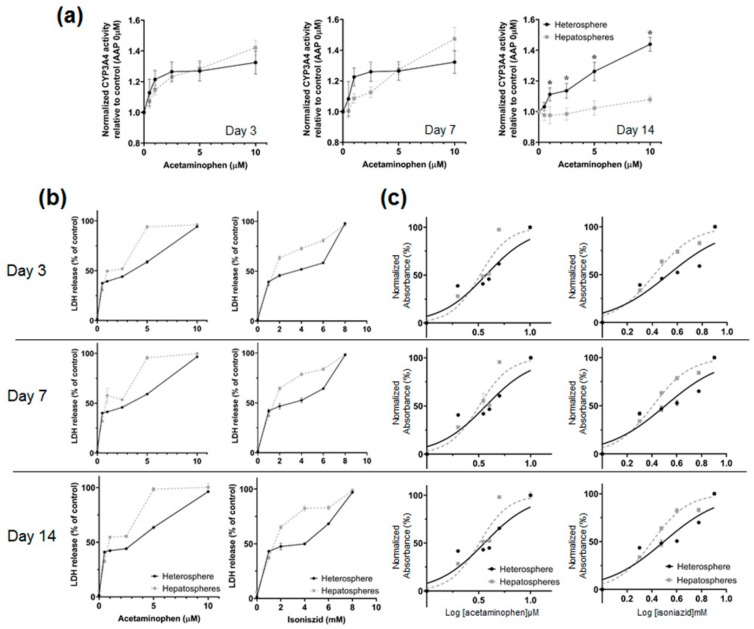
(**a**) Luminescence assay of cytochrome P450 activity in the hepatospheres and heterospheres after AAP treatment. Cytochrome P450 enzyme activity was detected according to the luminescence emitted upon addition of a detection reagent; the degree of luminescence emitted was measured using a luminometer. (**b**) The assessment of cell death using the LDH assay. The hepatospheres and heterospheres were treated with AAP and INH for 24 h. Data are depicted as the percentage of the total LDH amount. All values are showed as the mean ± SE of four independent experiments. * *p* < 0.05 (**c**) IC_50_ Determination curve.

The metabolic activity of hepatocytes was determined by monitoring the enzymatic activity of CYP3A4 over 14 days (Figure 6a). A luminescence assay showed stable CYP3A4 enzymatic activity in the heterospheres. The CYP3A4 activity was significantly higher in the heterospheres than in the hepatospheres on day 14. To examine the potential of the heterospheres for toxicity testing under flow culture conditions, we evaluated the cytotoxicity using the endpoint LDH release in the heterospheres and hepatospheres (Figure 6b). The IC_50_ values for AAP and INH after 24 h of exposure were calculated from the dose–response curves of the two compounds (Table 1). In this culture system, AAP and INH were used to investigate the optimized perfusion system of the hepatocyte spheroids for drug toxicity testing. The heterosphere and hepatosphere cultures on days 3, 7, or 14 were treated with AAP and INH for 24 h, and the IC_50_ values were determined using the LDH release assay. The IC_50_ values of the heterosphere cultures after AAP treatment on days 3 (2.23 μM), 7 (2.11 μM) and 14 (2.15 μM) were higher than those of the hepatosphere cultures on the corresponding days (day 3: 2.13 μM, day 7: 1.57 μM, and day 14: 1.02 μM). Similarly, the IC_50_ values of the heterosphere culture following INH treatment on days 3 (2.96 mM), 7 (2.81 mM) and 14 (2.66 mM) were higher than those of the hepatosphere cultures on the corresponding days (day 3: 2.35 mM, day 7: 1.8 mM and day 14: 1.25 mM) (Figure 6c). In the perfusion culture system, no significant difference was observed in LDH release following AAP and INH treatment on day 3 in both the spheroids. In contrast, the LDH release following the AAP and INH treatment varied greatly between the heterospheres and hepatospheres under flow culture conditions, on day 14. Based on these results, in the experimental period, the heterospheres showed a more stable range of IC_50_ values than the hepatospheres in flow culture conditions. Additionally, the heterospheres showed more stable metabolic activity than the hepatospheres. 

## 4. Discussion

Generally, conventional in vitro culture systems are known to lack hepatocyte functions; therefore, it is necessary to establish and make available a functional culture system that maintains their specific function. Many studies have demonstrated the maintenance and differentiation of hepatocytes cultured with a variety of non-hepatic cells [21,22,23]. The cell-associated and soluble factors were shown to keep the hepatocytes morphologically and functionally differentiated [24,25,26]. Although the co-culture systems consisting of hepatocytes and HSCs have been used to establish and improve in vitro liver models, these co-culture systems could not mimic actual cell quantity and the in vivo environmental situations. Since the HSC amounts to about 5–12% of the total cell population in the normal liver [1,27,28], HSCs may be used to improve the specific functions of hepatocytes. Thus, in the present study, we established co-culture systems containing hepatocyte and HSCs at a ratio of 9:1. Furthermore, to mimic the in vivo environment, we applied a flow rate in our system. We demonstrated that a flow-based co-culture system may be successfully used to maintain the viability and spheroidal shape, improve the secretion of albumin and urea, and increase the expression of hepatocyte-specific genes and metabolic activity. There are close relationships between the hepatocytes and hepatic stellate cells. On the one hand, HSCs are known to be the major production cells of the extracellular matrix, and secretory cells of numerous mediators in the liver [8,9,10]. They also assist in sustaining hepatocyte-specific functions and the formation of aggregates through cell–cell interactions in the in vitro culture as reported by Wong et al. [29]. Thus, as HSCs play a critical role in the maintenance of hepatocyte phenotype and function, it is necessary to establish a flow-based co-culture system containing HSCs, to maintain the liver-specific functions of hepatocytes. 

After achieving high and stable liver-specific functions and viability rates of spheroids in flow conditions, the spheroids cultured in flow-based chips were tested for their applicability in acute and chronic drug toxicity and metabolic function studies, using various concentrations of drugs. AAP, a painkiller, is a widely used pharmaceutical drug. It is metabolized via several cytochrome P450 isoforms, including CYP3A4, CYP2E1 and CYP1A2, to the reactive intermediate N-acetyl-*p*-benzoquinone imine (NAPQI) [30]. The optimal CYP enzymatic activity in hepatocytes is critical for the liver to conduct the detoxification of a wide range of toxins and drugs. We focused on the major class of CYP enzymes, CYP3A4, as these enzymes typically mediate the metabolism of pharmaceuticals. Our results showed that the activity of the CYP enzymes in the AAP-treated heterospheres was higher than that of the AAP-treated hepatospheres on day 14 (Figure 6a). These increases in xenobiotic gene expression and CYP activity in the co-culture model may be suitable to study drug–drug interactions mediated by CYPs. 

Additionally, this increased metabolic competence may improve the predictive power of in vitro 3-D hepatotoxicity screening. Recently, toxicity tests have been actively conducted using an in vitro hepatocyte culture system [31,32]. The liver is the major source of metabolism and drug biotransformation; thus, the toxicological and pharmacological testing of hepatocytes is important [33]. Using our spheroids cultured in a flow-based system, heterospheres and hepatospheres were exposed to different concentrations of AAP and INH for 14 days. As shown in Figure 5b, the drug sensitivity was concentration dependent. The increase in the LDH release upon treatment with various concentrations of AAP and INH was more constant in hepatospheres than in the heterospheres. Furthermore, the IC_50_ values of the heterospheres were more stable than those of the hepatospheres (Table 1). This implies that interactions between hepatocytes and HSCs may influence the process of different mechanisms of drug toxicity in the co-culture system. The heterospheres in the concave flow culture system have better cellular functions potentially through more in vivo-like complex adaptive response to chemical exposure. The co-culture model in the concave flow culture system showed that the heterospheres allow long-term and more stable in vitro hepatotoxicity prediction than the hepatospheres.

Over the years, many hepatocytes in vitro culture models have been emerged to allow the investigation of the harmful effects of chemicals and drugs. Consequentially, significant advancements have been made to improve conventional in vitro culture models, which maintain hepatocyte-specific functions, and may accurately predict in vivo response to toxic substances. Immortalized cell lines [34] and isolated hepatocytes [3,35,36] are extensively used in current times. These models, however, lack the ability to provide a long-term culture without cell necrosis or de-differentiation. Additionally, the loss of viability and decrease in liver-specific function and gene expression are common disadvantages of these models. Thus, heterospheres and hepatospheres produced using concave flow chips could be promising culture models for toxicology studies.

Heterospheres contain more HSCs, just like the liver, than that normally used in hepatospheres. Furthermore, our study showed that the co-culture system closely resembles the in vivo environment under flow culture conditions. Thus, the flow-based, co-culture system described here provides a reproducible in vitro environment by controlling cell proportion and flow rate, and it could be a promising long-term culture model.

## 5. Conclusions

In this study, we used our optimized, flow-based, co-culture system identifying the effect of HSCs, on hepatocyte function and viability. These results from the heterosphere culture indicated that it may be appropriate for long-term culture of hepatocytes. In addition, the use of flow-based in vitro systems in toxicity test has potentially many advantages, such as a decrease in animal numbers, reduced cost of animal maintenance and care, reduced quantity of chemicals needed for the test; moreover, in vitro system-based methods are less time intensive and offer increased throughput for evaluating multiple chemicals. Although additional in vitro researches may be required to acquire a better understanding of the beneficial effects and limitations of the co-culture system, our flow-based co-culture system provides a helpful and efficient platform for hepatocyte culture and toxicity tests in the in vitro microenvironment.

## Figures and Tables

**Table 1:** IC_50_ values for AAP and INH in the hepatosphere and heterospheres.

IC_50_ Values	Day 3		Day 7		Day 14	
	Heterosphere	Hepatosphere	Heterosphere	Hepatosphere	Heterosphere	Hepatosphere
IC_50_ of acetaminophen (µM)	2.23	2.13	2.11	1.57	2.15	1.02
IC_50_ of isoniazid (mM)	2.96	2.35	2.81	1.80	2.66	1.25
